# Universality of Rank-Ordering Distributions in the Arts and Sciences

**DOI:** 10.1371/journal.pone.0004791

**Published:** 2009-03-11

**Authors:** Gustavo Martínez-Mekler, Roberto Alvarez Martínez, Manuel Beltrán del Río, Ricardo Mansilla, Pedro Miramontes, Germinal Cocho

**Affiliations:** 1 Instituto de Ciencias Físicas, Universidad Nacional Autónoma de México, Cuernavaca, Morelos, México; 2 Centro de Ciencias de la Complejidad, Universidad Nacional Autónoma de México, Distrito Federal, México; 3 Instituto de Física, Universidad Nacional Autónoma de México, Distrito Federal, México; 4 Centro de Investigaciones Interdisciplinarias en Ciencias y Humanidades, Universidad Nacional Autónoma de México, Distrito Federal, México; 5 Facultad de Ciencias, Universidad Nacional Autónoma de México, Distrito Federal, México; Harvard University, United States of America

## Abstract

Searching for generic behaviors has been one of the driving forces leading to a deep understanding and classification of diverse phenomena. Usually a starting point is the development of a phenomenology based on observations. Such is the case for power law distributions encountered in a wealth of situations coming from physics, geophysics, biology, lexicography as well as social and financial networks. This finding is however restricted to a range of values outside of which finite size corrections are often invoked. Here we uncover a universal behavior of the way in which elements of a system are distributed according to their rank with respect to a given property, valid for the full range of values, regardless of whether or not a power law has previously been suggested. We propose a two parameter functional form for these rank-ordered distributions that gives excellent fits to an impressive amount of very diverse phenomena, coming from the arts, social and natural sciences. It is a discrete version of a generalized beta distribution, given by *f*(*r*) = *A*(*N*+*1-r*)*^b^*/*r^a^*, where *r* is the rank, *N* its maximum value, *A* the normalization constant and (*a*, *b*) two fitting exponents. Prompted by our genetic sequence observations we present a growth probabilistic model incorporating mutation-duplication features that generates data complying with this distribution. The competition between permanence and change appears to be a relevant, though not necessary feature. Additionally, our observations mainly of social phenomena suggest that a multifactorial quality resulting from the convergence of several heterogeneous underlying processes is an important feature. We also explore the significance of the distribution parameters and their classifying potential. The ubiquity of our findings suggests that there must be a fundamental underlying explanation, most probably of a statistical nature, such as an appropriate central limit theorem formulation.

## Introduction

During the past decade or so, a considerable amount of research has been devoted to power law behaviors, particularly with regard to complex networks [1], [2]. However, when real data is analyzed, in most of the cases the power law trend holds only for an intermediate range of values; there is a power law breakdown in the distribution tails [3], [4]. Both the breakdown point and the tail functional forms are of interest [5]. Several explanations have been provided for this phenomenon, such as finite size effects (e.g. insufficient data for good statistics) [6], [7], [8], network dilution, network growth constraints [3], [7] and different underlying dynamical regimes, leading to power law corrections (sometimes referred to as scaling corrections) in the form of exponential, Gaussian, stretched exponential, gamma and various types of extreme value distributions [9], [10]. In this work we focus on rank-ordered distributions, often related to cumulative distribution functions, which show the way in which a given property of a system is ordered decreasingly according to its importance (rank). Our main result is that a surprising amount of situations follow a two parameter distribution which incorporates the product of two power laws defined over the complete data set, one measured from “left to right” and the other from “right to left”. The fit holds for the full range of values, tails included, with correlations that rival with, or generally improve on, power law correction schemes proposed in the literature.

In our work a functional universality is revealed for rank-ordered distributions, encompassing apparently unrelated phenomena coming from music, painting, ecology, urbanism, neuroscience, genetics and social networks, amongst others. In the following we develop a phenomenology based on a selection of the vast number of cases where we have encountered this functional form. Prompted by some of these observations we implement a conflicting dynamics model that generates this distribution and contributes to the identification of relevant underlying features of processes leading to it, as well as to a characterization of its parameters. From our exploration we also detect that the convergence of multiple heterogeneous processes appears to be an important factor. Overall, our findings suggest that there must be a deep underlying explanation, possibly of a statistical nature.

## Results

### Phenomenology

Rank-ordered relations show how given property of a process decreases [1], [2], [7], [8]. A well studied instance of this is the so called Zipf law [11] which originally referred to the frequency with which words are used in a specific language. Zipf showed that the logarithm of the frequencies with which words appear in the novel Ulysses by James Joyce, when plotted in decreasing order against the logarithm of their rank, fall on a straight line with slope *−1*, thus indicating a power law behavior. However, in general, this straight line behavior with negative slope holds only within an intermediate rank range [12], [13]. Here we show that for this phenomenon of common occurrence, the power law corrections have themselves universal features, further more, a surprising amount of systems of very diverse nature which do not follow power laws at all, present a common statistical behavior expressed by a generic rank-ordered distribution function.

**Figure 1:**
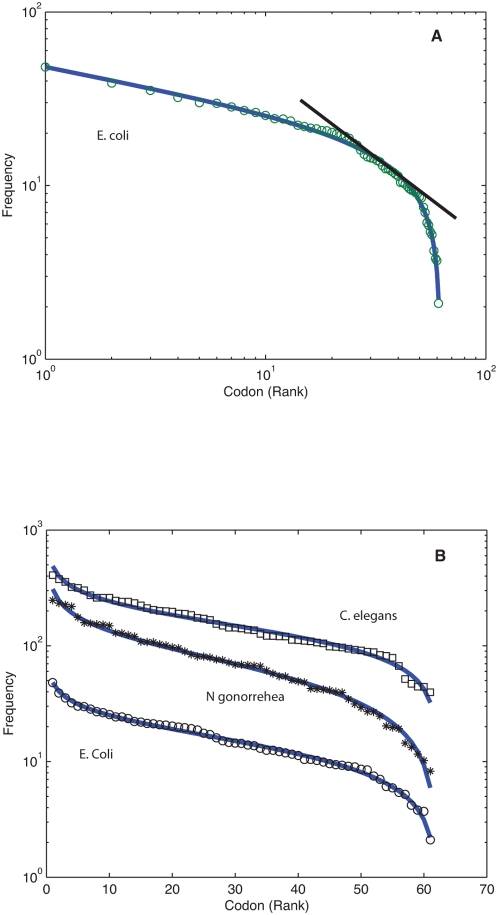
Frequency-rank in genetic sequences. (A) Log-log plot of the frequency, in descending order, with which the codons appear in the genome of E. coli. The bold line is the discrete generalized beta distribution (DGBD) fit with exponents and squared correlation coefficient (*a*,*b*,*R^2^*) = (*0.25*, *0.50*, *0.99*). The straight line is included as a guide to the eye of a power law behavior within a restricted range. (B) Semi-log plot of the frequency-ordered codons of the genomes of *C. elegans*, *N. gonorrehea* and *E. coli*. Solid lines are the fits with (*a*,*b*, *R^2^*) = (*0.28*, *0.38*, *0.98*), (*0.31*,*0.65*, *0.99*) corresponding to the first two, values for *E coli* are given in (A). Frequencies for *N. gonorrhea* have been multiplied by a factor of 5 and those of *C. elegans* by 10 in order to avoid overlaps.

As a starting point we consider systems consisting of symbols arranged sequentially such as codons (nucleic acid triplets that code for amino acids) in genes or notes in musical scores. In Fig. 1A we show a log-log plot of the frequency with which the 61 possible codons (stop codons excluded) appear in the coding genetic sequences of the bacterium *Escherichia coli*, plotted in decreasing order from the most common to the least common one. Notice the power law like behavior in the intermediate range and the steeper finite size decay for the less frequent occurrences. If we plot the same data in a semi-log representation, together with the codons of the genes of *Nesisseria gonorrhea* and the worm *Caenorhabditis elegans*, we obtain the sigmoid type graphs shown in Fig. 1B. In this representation the full data range is given equal standing. The form in the semi-log graphs in the region to the left of the inflexion point is suggestive of a logarithmic decay, while the one to the right brings to mind a logarithmic behavior with the independent variable measured from right to left; we therefore test the pertinence of using a functional form incorporating the above mentioned features as a fit for the data, namely:
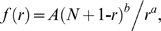
where *r* is the rank value, *N* its maximum value, *A* a normalization constant and (*a*, *b*) two fitting exponents. This expression is a discrete version of the continuous random variable generalized beta distribution and we shall refer to it from now on as DGBD [14], [15]. The bold curves in Fig. 1 show that the functional form is a very good choice. The square of correlation coefficients, *R^2^*, determined by a log-log multiple linear regression, lie between 0.98 and 0.99. We have obtained similar results, for tens of organisms covering archea, bacteria and eukaryotes, both for amino acid and codon distributions.

**Figure 2:**
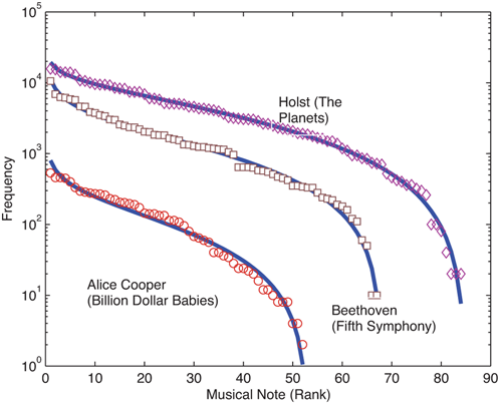
Frequency-rank distributions for musical scores. Plot of the occurrence of musical notes, ordered decreasingly, in the scores of Holst's “The Planets”, Beethoven's first movement of the “Fifth Symphony” and Alice Cooper's “Billion Dollar Babies”. Solid lines are DGDB fits with (*a*,*b*,*R^2^*) = (0.23, 1.54, 0.988), (0.42,1.25, 0.987), (0.71, 1.06, 0.978).

If we now look into the arts, we have that notes in musical scores provide another example of sequences of symbols where rank frequency DGBD are encountered. Fig. 2 shows compositions by Beethoven, Holst and the rock band Alice Cooper. Again correlation coefficients are very high, with *R* ^2^ above 0.98, notice that fit is very good for the whole range of values. The analysis of more than 1800 compositions shows that this type of behaviour is recurrent. Furthermore, fitting parameters (*a*,*b*) appear to be sensitive to whether the musical composition is in a minor or mayor scale [16].

**Figure 3:**
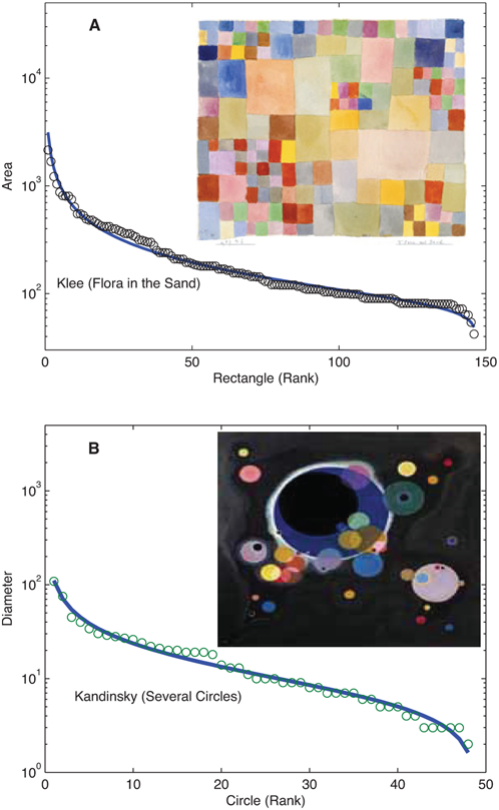
Size-ordered distributions in abstract paintings. (A) Plot of rectangle relative sizes in arbitrary units shown in decreasing order appearing in Klee's painting “Flora in the sand”. Bold line is the DGBD fit with (*a*,*b*, *R^2^*) = (0.70, 0.14, 0.999. (B) Plot of circle relative areas expressed in arbitrary units present in Kandinsky's “Several Circles” arranged in decreasing order, here the bold line fit has (*a*,*b*, *R^2^*) = (0.62, 0.32, 0.978).

Still in the arts, keeping in mind that the frequency of occurrence of a note is in some sense related to the “length” occupied in a given score, we determine the area occupied by specific geometric motifs in abstract painting, such as rectangles in canvases by Paul Klee and Piet Mondrian or circles in works of art by Kandinsky. We then order these determinations as rank-size distributions and adjust DGB distributions. In Fig. 3 we show fits for Klee's “Flora on Sand” and Kandinsky's “Several Circles” respectively, again with *R^2^* values above 0.98.

**Figure 4:**
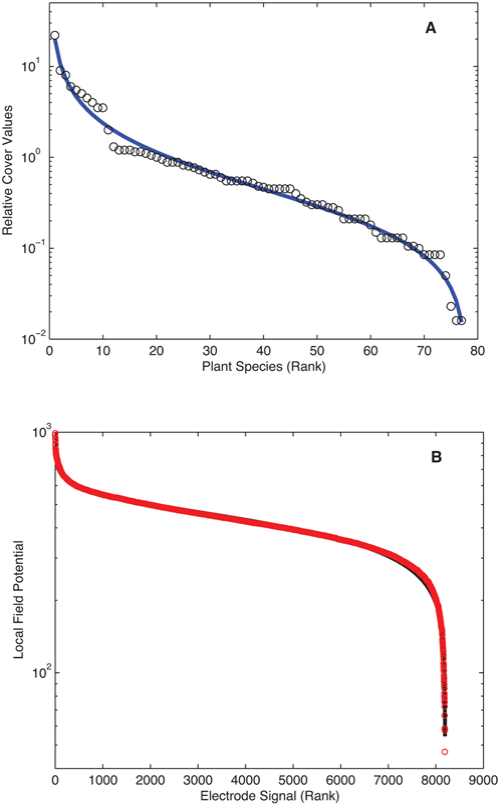
Rank-ordered distributions in biological systems. (A) Plot of the relative area occupied by different species in abandoned fields of Ilinois over a span of 40 years [17]. For this case (*a*,*b*,*R^2^*) = (0.88, 0.76, 0.98). (B) Local field potential measurements of cat cerebral cortex taken every 4 ms in an awake state, total of 8192 data points plotted in decreasing order [18] (a,b,R^2^) = (0.08,0.25,0.98).

An environmental case is shown in Fig. 4A for plant species diversity in old-field successional ecosystems. Here the rank ordered data refer to the relative cover values of the plant species encountered in 40 year old abandoned fields in Southern Illinois [17].

Fig. 4A is related to neurophysiology [18], it shows that rank ordered local field potential measurements in cat cerebral cortex during natural wake states follow very closely a DGBD, (*a*,*b*,*R^2^*) = (*0.081*, *0.239*, *0.97*). When slow wave sleep states (SWS) are considered the fit worsens while rapid-eye-movement (REM) periods resemble awake state results.

**Figure 5:**
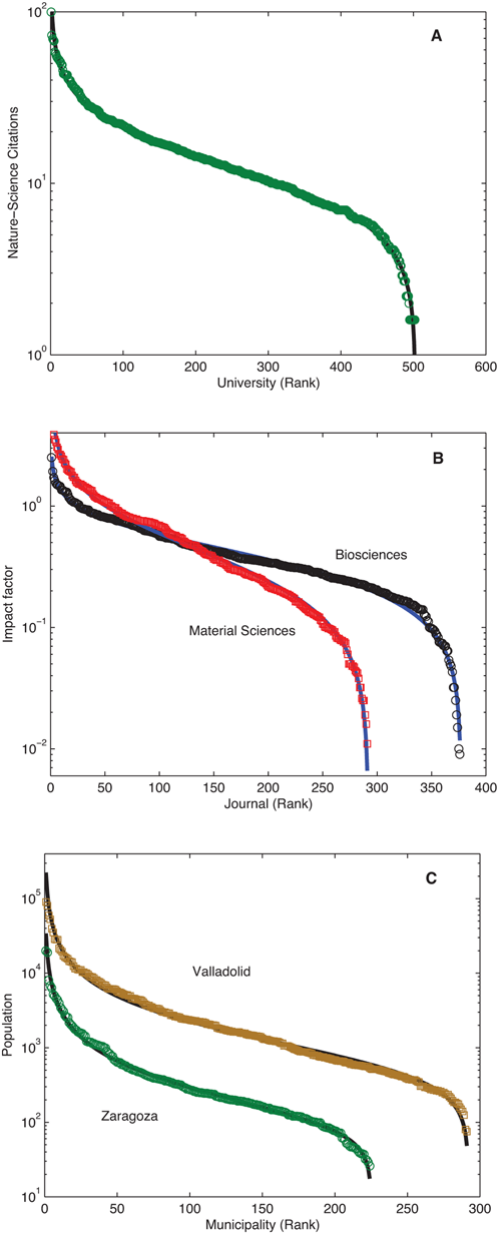
Rank-ordered distributions in social phenomena. (A) Academic ranking of world Universities [19] based on the number of publications in Nature and Science,(*a*,*b*,*R^2^*) = (*0.37*,*0.43*,*0.99*). (B). Bioscience and material science journals ordered by impact factor [20] (*a*,*b*,*R^2^*) = (*0.59*, *0.83*, *0.99*),(*0.51*,*0.75*,*0.99*) respectively. (C). Population of the municipalities of the Spanish provinces of Zaragoza and Valladolid [21] (*a*,*b*,*R^2^*) = (*0.95*, *0.54*, *0.99*), (*0.98*,*0.42*,*0.99*) *respectively*.

A rank ordered distribution related to society, is presented in Fig 5A for the world wide classification of universities according to their number of contributions to the journals Nature and Science between 2002 and 2006 [19]. Here the square of the correlation coefficient is 0.99. In Figs. 5 B,C we show fitting results with *R^2^* above 0.99 for two other examples of social bearing: the journal impact factor ranking [20] and population ordered municipalities of Spanish provinces, respectively [21].

**Figure 6:**
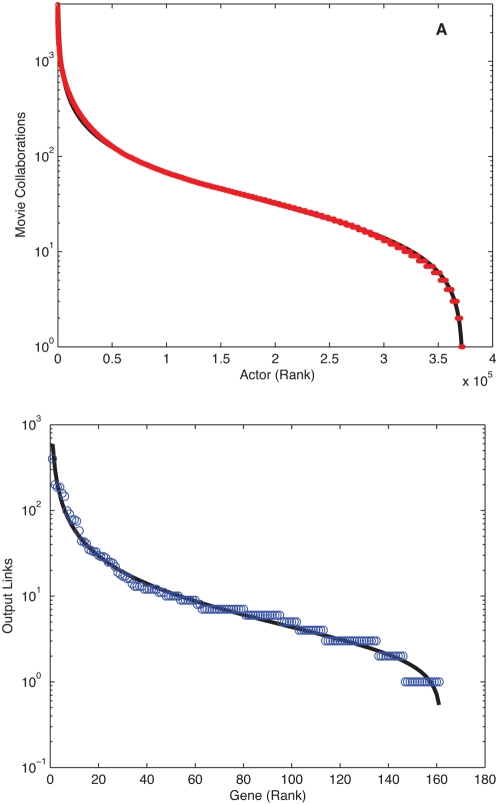
Rank-ordered distributions in networks. (A) Movie actor network based on the Internet Movie Database (c.f. http://www.nd.edu/~networks) containing 372,794 actors linked by movie collaborations (*a*,*b*,*R^2^*) = (*0.71*,*0.61*,*0.99*). (B) *E. coli* regulatory network nodes ordered by the number of output links based on the data of reference [22].

As DGBD network examples we show the movie actor collaborative distribution [1] (see Fig. 6A) and the rank-size distribution of the out-bound links of the *E. coli* genetic regulatory network [22] (see Fig. 6B). In the former each node is an actor, and two actors are connected if they were cast in the same movie. Though this network has been extensively studied in the literature and good results for the connectivity probability have been found with alternative two parameter distribution functions [23], our DGBD fit reaches remarkable accuracy, reproducing qualitative features.

For comparative purposes in Table 1 we show the values of (*a*,*b*,*R^2^*) for several representative examples of diverse nature, some of them taken from previous figures.

**Table 1:** Fitting parameters *a*, *b* and correlation coefficient *R^2^* for diverse systems.

	*a*	*b*	*R^2^*
Letters in English	0.18	1.31	0.97
Musical Notes in Haendel's Messiah	0.56	1.46	0.98
Area of Motifs in Malevich's Airplane Flying	1.1	0.57	0.98
Old-field Ecosystems	0.88	0.76	0.98
Local Field Potential in Cat Cerebral Cortex	0.08	0.24	0.97
Crashes of U.S. Stock Exchange	3.56	0.11	0.98
E.coli Genetic Regulatory Network	0.99	0.39	0.98
Movie Actors Network	0.71	0.61	0.99
Academic Ranking of World Universities	0.37	0.43	0.99
Biosciences Journal Impact Factor	0.59	0.83	0.99
Mexican State Population	0.44	0.68	0.99
Zaragoza Municipality Population	0.95	0.54	0.99
Valladolid Municipality Population	0.98	0.42	0.99
Chinese Province Population	0.14	0.98	0.99
Highway Distance from Guanajuato to Major Mexican Cities	1.52	3.87	0.99

Data sources are for: letters in the Concise Oxford Dictionary [29] (similar results hold for other 25 languages we have looked into), musical notes come from the musical score, relative area occupied by different species in abandoned fields of Illinois [17], journal impact factor in biosciences and material sciences journals [30], Mexican state population [31], Chinese population [32], Zaragoza and Valladolid municipality population, Mexican highways [31].

### Model

**Figure 7:**
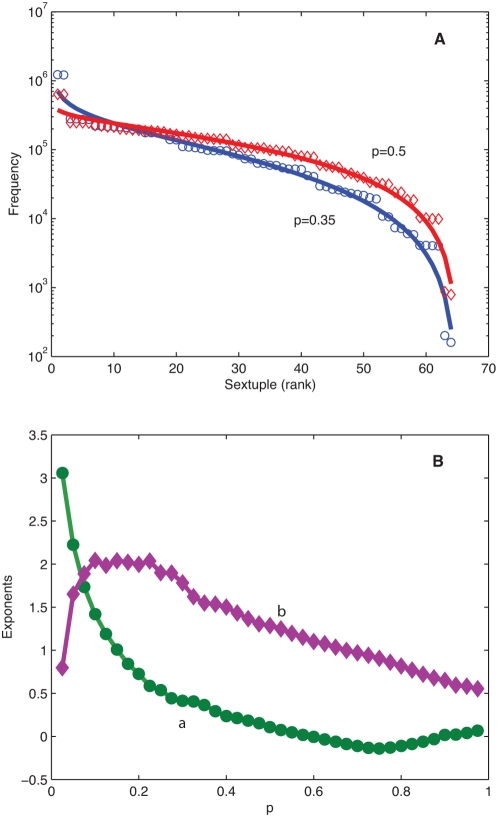
Frequency-rank distributions of sextuples generated by an expansion-modification algorithm. (A) Data is generated by the algorithm described in the text. Circles are determined with a modification probability *p* = *0.35*, the corresponding solid line is the DGDB fit with (*a*,*b*,*R^2^*) = (*0.36*,*1.55*,*0.96*). For the rhomboids *p* = *0.5* and (*a*,*b*,*R^2^*) = (*0.11*,*1.28*,*0.96*). (B) shows the variation of the parameters (*a*,*b*) with probability *p*.

The material presented so far is only a sample of the variety of situations where we have encountered a rank ordering statistical behavior following closely the DGBD. This poses the challenge of unraveling mechanisms or identifying characteristics that may contribute to some understanding of these findings [24]. Prompted by our analysis of genetic sequences, as a step in this direction we work with an expansion-modification dynamics introduced by Li [25], [26], where two processes converge, one related to permanence the other to change. This model incorporates basic elements of a neutral evolution scheme in which the main mechanisms for change in sequences are duplications and point mutations. The simplest Boolean realization of this scheme is the following: *i)* consider a system with variables that can only take two values, say *0* and *1*; *ii)* initiate a process with either one of these values by applying with probability *p* the modification (point wise mutation) rule: *0* goes to *1*, or *1* goes to *0*, and with probability *1-p* the expansion (duplication) rule: *0* goes to *00* or *1* goes to *11*, *iii)* generate a growing sequence of zeros and ones by a repeated application of the preceding algorithm. After a large number iterations of this algorithm, the statistical behavior of the ensuing sequence can be tested by looking into the frequency-rank of *n-tuples* (non-overlapping groupings of *n* consecutive elements). Here we have implemented a slight variation of the algorithm described above which enhances expansion, namely *0* goes to *000* and 1 goes to *111*, both cases with probability *1-p*. This makes it somewhat more “realistic” in genetic terms. In practice we start with a 0 or 1 seed chosen with probability 0.5. After 128000 iterations the out coming sequence is treated as an initial condition and further iterated 10^6^ times. The frequency with which non-overlapping sextuplets occur is then averaged over 10 realizations of this process. Fig. 7A shows this average frequency in decreasing order for two values of the modification probability *p*, as well as the corresponding DGBD. In Fig. 7B the values of the fitting parameters *a* and *b* are plotted against *p*. For *p* very small, *a*>*b*, point mutations are rare and expansion is favored, leading to extended intervals of zeros or ones; as *p* grows *a* and *b* eventually meet since *a* decreases and *b* increases. Above this threshold value *p_th_*, *a*<*b* and the higher likelihood of point mutations induces more disorder. From this perspective *a* is related to permanence and *b* to change. Eventually, for values of *p* sufficiently large, intervals of alternating zeroes and ones start to dominate, reducing the degree of disorder and decreasing the value of *b*, which however continuous to be greater than *a*. Modifications of this model by introducing independent probabilities for mutation and modification, different expansion rates, as well as delays for mutation application, all produce sequences with good DGBD. Threshold values are sensitive to these changes and may even be absent.

This behavioral pattern is further reinforced by looking into families of deterministic discrete time evolution rules of continuous variables (mappings) where permanence relates to regular (laminar) behaviors and change appears from chaotic (turbulent) dynamics. For both the discrete models of the previous paragraph and these continuous models it can be shown that the point *a* = *b* signals a disorder transition. In the first case this coincides with the end of scale invariant regions [25], in the second it marks the onset of maximum entropy.

## Discussion

**Figure 8:**
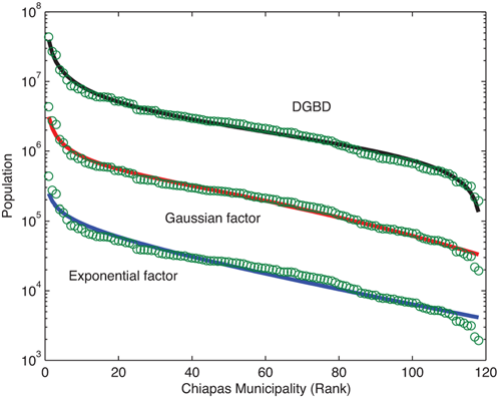
Two parameter fits for rank ordered data. The figure shows three fits for the population of the municipalities of the Mexican state of Chiapas [26] plotted in decreasing order. The bottom set of points corresponds to the original data, the other two sets have been obtained by successively multiplying by 10 in order to distinguish the behavior of the each fit. The top fit is the DGDB distribution, the middle one corresponds to a power law multiplied by a Gaussian factor and the bottom is a power law multiplied by an exponential factor. All fits have two adjustable parameters and produce good values for *R^2^*, in the neighbourhood of 0.97. Notice however that the DGBD curve reproduces more successfully the overall form of the data, particularly at the two extremes.

Overall we have encountered a universal behavior defined in terms of a functional relation for rank ordered distributions that holds accurately along the whole rank range for an impressive amount of phenomena of very diverse nature. It is not surprising that this expression goes beyond power laws since it is a two parameter relation that reduces to a power law when one of them is zero. Special interest arises when power laws require corrections due to finite size effects or other considerations. Under these circumstances they have often been modified by the inclusion of one or more additional parameters, e.g. Gaussian or exponential cut-offs. In most of the examples we have studied, though this type of correction often improves fits, our DGBD is quantitatively and above all qualitatively more satisfactory (see Fig. 8 for an example). Our main point is that, regardless of the presence of a power law, we have found a generic behavior previously not identified.

With regard to the meaning of the DGBD parameters, in some instances the exponent *a* can be related to behaviors generating power laws, as is the case of scale invariance in turbulence in the so called inertial range where energy is transferred between different scales at the same rate, while *b* seems to be associated with chaotic, disordered fluctuations, for example the dissipative range for turbulence [27]. The DGBD manages to encompass both types of regimes as well as their crossover. Further understanding of the exponents comes from our expansion-modification study where a conflicting dynamics leads to the DGBD. The expansion component which preserves a given trend is associated with *a*, on the other hand the modification part favors change and is related to *b*. Though we have shown that these conflicting permanence-change processes can produce DGBD, we are in no position to consider them as a requirement. On occasions we have perceived that parameters relations hold for certain instances, for example the for the musical notes frequencies *a*<*b* in general, while for network connectivity related situations *a*>*b* is encountered more often. However, the role of exponents *a* and *b* as universality classifying parameters, as for example in critical phenomena [28], remains be investigated in further detail.

Our findings are most revealing when both parameters *a* and *b* are non-negligible and not too disparate. This usually happens for the social phenomena we have explored and which present some the most impressive fits. Based on these examples, it appears that DGBD fits are at their best when dealing with situations that result from the convergence of multiple heterogeneous processes. These are most probably weakly correlated, for example as a result of constrictions. Such considerations are in accordance with the old-field relative occupation studies previously mentioned [17] where data has been collected for various types of vegetation; we have found that the statistical behavior of each type considered separately follows less convincingly the DGBD than the integration of them shown in Fig 4A. From the above, it seems also worthwhile to analyze the role of constrictions in the art and music examples. Additionally, consideration of phenomena with processes operating at different scales, as well as multinomial multiplicative processes [24] seem promising for a better understanding of our observations. All in all, the ubiquity of our findings suggests that there ought to be a fundamental underlying explanation of a statistical nature, such as a central limit theorem extension or reformulation for the class of systems we have been encountering.
